# ASO Author Reflections: The Changing Role of Gene Expression Profiling in ER+/HER2− Breast Cancer

**DOI:** 10.1245/s10434-019-07955-y

**Published:** 2019-10-24

**Authors:** Julia E. C. van Steenhoven, T. van Dalen

**Affiliations:** 1 Department of Surgery, Diakonessenhuis Utrecht, Utrecht, The Netherlands; 2 Department of Pathology, University Medical Center Utrecht, Utrecht University, Utrecht, The Netherlands

## Past

International guidelines increasingly question the benefit of adjuvant chemotherapy in selections of estrogen receptor-positive (ER+)/human epidermal growth factor receptor 2-negative (HER2−) breast cancer patients.^1^ At the same time, and in the same selection of patients, gene expression profiles (GEPs), such as the 70-gene signature (70-GS), are used as a means to better guide chemotherapy decisions. Previous studies demonstrated that use of the 70-GS was associated with a significant reduction in chemotherapy administration in patients with ER+/HER2− disease of low or intermediate malignancy grade without overt lymph node metastasis (≤ Nmi).^2^ In the present study, we assessed recent trends in the administration of adjuvant chemotherapy in patients eligible for GEPs and evaluated the role of the 70-GS on chemotherapy administration in lymph node-negative (N0) and lymph node-positive (N+) breast cancer patients.

## Present

At a nationwide level, the overall administration of adjuvant chemotherapy in patients eligible for GEP use decreased from 49 to 23%, while 70-GS use increased from 24 to 51%.^4^ The decline in chemotherapy administration occurred without a change in national breast cancer guidelines,^3^ but coincided with recent international guideline recommendations.^1^ In contrast to previous studies,^2^ the observed decline in chemotherapy use between 2013 and 2016 occurred in N0 patients irrespective of 70-GS use, and mainly in N0 patients in whom the 70-GS was not used. In contrast, in N+ patients, use of the 70-GS was strongly associated with a decreased likelihood of receiving chemotherapy throughout the study period.^4^ In the present study, the effect of age on the decision to administer adjuvant chemotherapy was remarkable. In patients < 50 years of age and 50–59 years of age, the 70-GS was strongly associated with a decreased probability of receiving chemotherapy, whereas in older patients (60–69 years), a reversed association was observed.^4^

## Future

The present study reflects a growing restraint of clinicians to administer chemotherapy in selections of ER+/HER2 patients. In clinical low-risk (N0) patients, this leads to less patients receiving chemotherapy irrespective of 70-GS deployment. This is in line with the results of the recently published MINDACT trial, showing no additional value of the 70-GS in clinical low-risk patients.^5^ Efforts should be made to better delineate the category of ER+/HER2− patients who are to be considered as clinical ‘low risk’ patients and who will not be candidates for chemotherapy use or 70-GS deployment. On the other hand, in categories of patients who are still considered as ‘clinical high risk’, e.g. younger women and node-positive patients, 70-GS use has an important impact in terms of a 70-GS use-associated decrease in the proportion of patients treated with adjuvant chemotherapy. In these latter patient categories, gene expression profiling should be more strongly advocated in order to avoid overtreatment. Long-term follow-up of ongoing trials into gene expression profiling will be crucial to corroborate this de-escalating approach.
